# Compound Pollen Protein Nutrient Increases Serum Albumin in Cirrhotic Rats

**DOI:** 10.4021/gr240e

**Published:** 2010-11-20

**Authors:** Hong Bo Shi, Ming Kong, Gong Chen, Jun Zhao, Hong Lin Shi, Yu Chen, Frank G Rowan

**Affiliations:** a Beijing Municipal Institute of Liver Diseases, Beijing Youan Hospital, Capital Medical University, China; b Department of Experimental Medicine, McGill University, Quebec, Canada

**Keywords:** Compound Pollen Protein, Albumin, Liver cirrhosis, Rat, Nutrition treatment, Liver regeneration, Noveliver

## Abstract

**Background:**

Malnutrition, especially protein-calorie malnutrition, is common in patients with liver cirrhosis. When in the status of malnutrition, the complications increase, liver function deteriorates, and the prognosis of patients with liver cirrhosis worsens. Hence, nutritional support and treatment is essential in patients with liver cirrhosis. Previous studies suggested that compound nutrition based on pollen can improve liver function, and can be a basic nutrient for patients with liver cirrhosis. However, the nutritional support based on pollen for malnutrition of cirrhotic patients needs to be further evaluated. In this study, we investigated the nutritional support of Noveliver, a new compound pollen protein nutrient, in the cirrhotic rats induced by carbon tetrachloride (CCl_4_).

**Methods:**

The cirrhotic rats induced by CCl_4_ were treated with Noveliver in different doses, and treated with a regular compound pollen nutrient, untreated cirrhotic rats and normal rats were used as controls. Serum albumin were measured before and after the nutritional treatment in each group. At the same time, liver function, cytokines and pathological changes were also determined.

**Results:**

In the second week of nutritional treatment, the levels of serum albumin in normal control group, low dose noveliver group, high dose noveliver group, compound protein pollen group and spontaneous recovery group were 35.67 ± 1.42, 33.07 ± 1.27, 32.27 ± 1.50, 30.53 ± 0.25, 24.53 ± 3.56 (g/L), respectively, the differences among the groups were significant (F = 14.007, *P* = 0.000); The levels of serum albumin in low dose Noveliver group, high dose Noveliver group and the compound protein pollen group were higher than that in the spontaneous recovery group (*P* = 0.000, 0.001, 0.003, respectively). In the second week of nutritional treatment, the serum levels of HGF in normal control group, low dose Noveliver group, high dose Noveliver group, compound protein pollen group and spontaneous recovery group were 101.55 ± 0.87, 94.62 ± 8.80, 98.94 ± 3.68, 78.77 ± 21.79, 39.52 ± 14.03 (pg/ml), respectively, the differences among the groups were significant (F = 11.12, *P* = 0.002); the levels of HGF in low dose Noveliver group, high dose Noveliver group and the compound protein pollen group were higher than that in spontaneous recovery group (*P* = 0.001, 0.000, 0.005). Histological results showed that the fibrosis in spontaneous recovery group was severer than those in low dose Noveliver group, high dose Noveliver group and compound protein pollen group.

**Conclusions:**

Our data show that the both the Noveliver and the compound pollen protein nutrient increase the serum albumin and ameliorate malnutrition in cirrhotic rats; the recovery of serum albumin might be related to the hepatic damage repair and liver regeneration.

## Introduction

Cirrhosis is one of the main causes of mortality among Chinese adults and is also one of the leading causes of death for middle-aged males. Hospitalization costs for cirrhosis related complications are estimated at $18,000 per episode of care, and 10% of admitted patients die [1]. If the medical treatment of liver cirrhosis depends only on drugs and no account of the basic nutritional support is taken, the effect of treatment is limited and insufficient [2]. Recently, nutritional support in the treatment of liver cirrhosis has attracted increasing attention [3].

Malnutrition is very common in patients with liver cirrhosis, especially in the protein-calorie malnutrition, which has many clinical features, such as thin, anemia, immunological function disorder, and decreased serum albumin, even the ascites and abdominal infection [4]. Madden et al found that in 100 cirrhotic patients, 53 were adequately nourished, 33 were moderately malnourished, while the remaining 14 were severely malnourished. A reduced nutritional status, i.e., protein and energy malnutrition, has prognostic significance resulting in an increased complications and mortality rate [5]. Lautz et al had a follow-up study in 123 patients with liver cirrhosis who were considered as potential candidates for liver transplantation, data showed that the mortality rate in patients with malnutrition was 35%, which was twice as great as patients without malnutrition (16%); patients with malnutrition had more complications such as ascites and upper gastrointestinal hemorrhage than those without malnutrition; 28% of malnutrition patients needed liver transplantation which were more than those without malnutrition (16%); the mortality rate after transplantation in patients with malnutrition (53%) were higher than that without malnutrition(16%) [6]. In another study, nutritional status of 1402 patients with cirrhosis (883 males and 519 females) was recorded between 1988 and 1989 by the Italian Multicentre Cooperative Project on Nutrition in Liver Cirrhosis. They found that malnutrition could reduce the survival rate in patients with liver cirrhosis, and the reduction in muscle mass could be an independent risk factor for prognosis evaluation in patients with liver cirrhosis (Child-Pugh Classes A and B) [7]. All these previous studies show that the nutritional support and treatment is necessary for the patients with liver cirrhosis.

Pollen is a natural material with rich nutrents. Pollen contains high quality protein (20% - 30%), carbohydrate (40% - 50%), fat (5% - 10%), minerals (2% - 5%), water (15% - 25%). In addition, pollen has 22 kinds of amino acids, 15 kinds of vitamins, 20 kinds of trace elements, and more than 80 kinds of biological materials such as enzymes, hormones, flavones and plenty of nucleic acids [8]. Previous studies of pollen in the treatment of liver disease were little and focused mainly on liver injury, whereas studies of pollen in liver cirrhosis were not reported. Gokhan et al found that bee pollen has antioxidant effects, and can lessen rat liver injury caused by carbaryl and propoxur [9, 10]. Norma et al also confirmed that pollen from mesquite (*Prosopis juliflora*, Leguminosae) can protect liver injury in bromobenzene-intoxicated mice [11]. Zheng et al concluded that bee pollen plays an important role in the protection against acute hepatic injury and the this effect is dose-dependent [12]. Sun et al found that rape bee pollen and its different extracts can reverse the liver damage caused by alcohol [13]. Hence the compound nutritional support based on pollen can protect liver, and may be a better material for liver nutritional support.

In this study, using nutritional treatment based on pollen in cirrhotice rats induced by CCl_4_, we examined changes of serum albumin and its characteristics before and after treatment. In addition, we observed liver function, cytokines and hepatic histology changes. We attempted to find new clues for nutritional support of compound pollen in the cirrhotic patients, and explored the possible mechanisms underlying.

## Materials and Methods

### Animals

Forty-five male SD rats, weighing from 180 - 200 g, provided by Animal Center of Academy of Military Medical Sciences. All animals were housed in a pathogen-free environment maintained at 12-hour dark and light cycles with temperature (22 - 24 °C) and humidity (30% - 40%) control. This study was approved by Ethics Committee of Beijing Youan Hospital, Capital Medical University.

### Compound pollen protein nutrients

Noveliver was developed by Myer Otec Research Institute on Hepatocyte Matrix, California, USA (No: 20100426). It is mainly composed of bee pollen, albumin, phospholipids and yeast rich in selenium. It was dissolved in distilled water and was fed to the animals in two dosages, low and high dose groups by 3.5 and 7 times adult clinical therapeutic dose. The control compound protein pollen was developed by Beijing Baihua Bee Product Limited Company (No: 20100512). It is mainly composed of bee pollen and bee grub, the dosage was the same as Noveliver. Compared with compound protein pollen, Noveliver was added selenium and folic acid on the basis of the pollen, the ratio of aromatic and branched-chain amino acid was adjusted, Noveliver was developed for malnutrition and complications in end stage liver diseases.

### Experimental design

**Figure 1:**
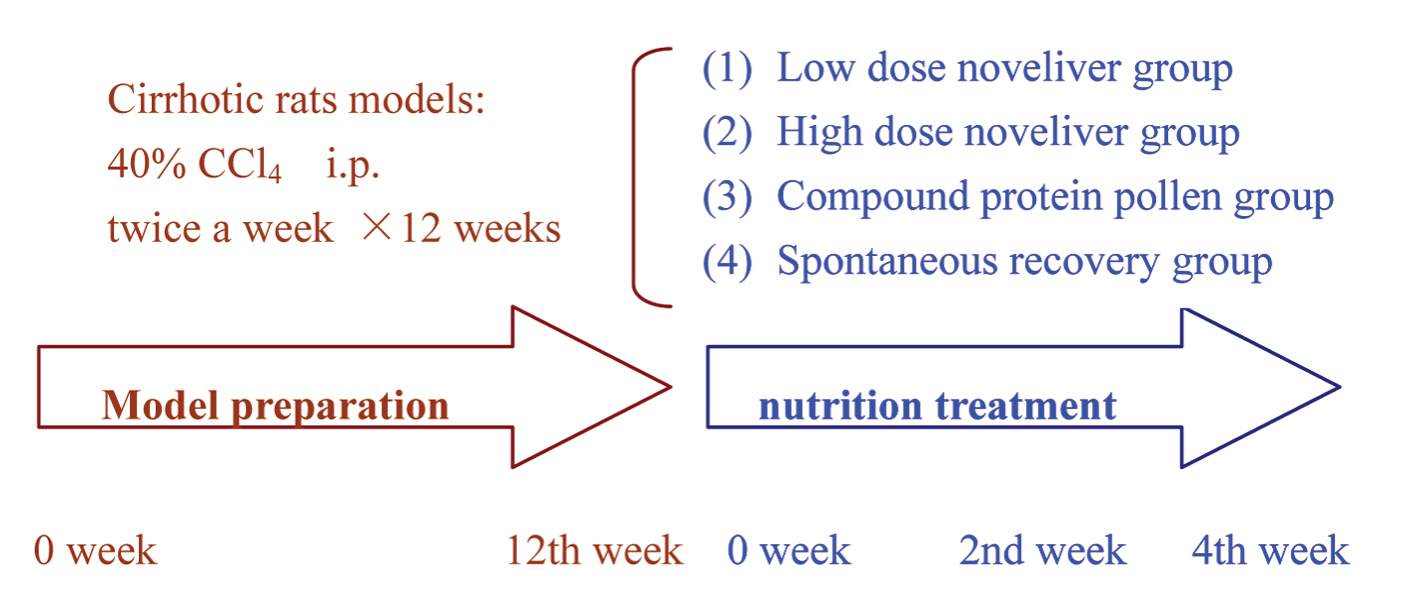
Illustration of rat model preparation and nutrition Treatment.

Forty-five rats were randomly divided into model group (n = 35) and normal control group (n = 10). The rats in model group were injected intraperitoneally mixture of carbon tetrachloride (CCl_4_, 40%) and oil (60%) by 0.2 ml per 100 g weight, twice a week for 12 weeks. Normal control group was injected same volume of saline solution in the same time and route as the model group. After 12 weeks, hematoxylin and eosin (HE) staining of liver tissue showed pseudolobuli formation which indicated the model was prepared successfully. In the 12th week, the normal control rats were all alive and continued normal food and drink, these rats were killed at 0, 2, 4 weeks of nutritional treatment, respectively. In the 12th week, 32 rats in the model group were prepared successfully, 4 of them were killed, and the rest were divided into four groups: (1) Low dose Noveliver group (n = 7): CCl_4_ injection discontinued at the 12th week and fed with low dose Noveliver twice a day, and killed in the 2nd and 4th week of nutritional treatment; (2) High dose Noveliver group (n = 7): CCl_4_ injection discontinued in the 12th week and fed with high dose Noveliver twice a day and killed in the 2nd and 4th week of nutritional treatment; (3) Compound protein pollen group (n = 7): CCl_4_ injection discontinued in the 12th week and fed with standard dose compound protein pollen twice a day, and killed in the 2nd and 4th week of nutritional treatment; (4) Spontaneous recovery group (n = 7): CCl_4_ injection discontinued in the 12th week and fed with same volume of distilled water as nutrient twice a day, and killed in the 2nd and 4th week of nutritional treatment, (Fig. 1)

### Collection and detection of samples

Rats were anaesthetized with chloral hydrate and fixed in supine position, abdominal cavity was opened and 10 - 15 ml of postcaval vein blood was collected from each rat. Whole liver was excised, the left lobe was fixed in 10% neutral buffered formalin for 24 hours, then the liver was embedded in paraffin, 4 µm thick sections were obtained with microtome. The rest liver tissues were put in saline solution and preserved in -80 °C freezer. The levels of albumin, ALT, AST, TBil, DBil in serum were detected in the Clinic Center of Beijing Youan Hospital with Automatic Biochemistry Analyzer.

### The levels of serum HGF and TGF-β1 by ELISA

The rat HGF and TGF-β1 ELISA kits were placed in room temperature (20 - 25 °C) for 15 - 30 minutes. All standards were prepared before the start of the assay procedure. The desired number of coated wells were secured in the holder, then added 5 µL of standards or samples in the appropriate well of the antibody pre-coated microtiter plate. A total of 200 µL of biotin labeled antibody was added to each well and mixed completely, the wells were covered and incubated for 30 minutes at 37 °C. Then the the microtiter plate was washed for 5 times. After final wash, plate was inverted and dried by hitting plate onto absorbent paper until no moisture appeared. A total of 200 µL of HRP conjugate was added to each well and mixed completely. The wells were covered and incubated for another 30 minutes at 37 °C. The microtiter plate was washed using the methods as above. A total of 100 µL of substrate TMB was added to each well and the wells were covered and incubated for 20 minutes at 20 - 25 °C, then 100 µL of stop solution was added to each well and mixed. The optical density was read at 450 nm using a microtiter plate reader within 30 minutes.

### Hematoxylin and Eosin staining protocol of liver tissue

The liver sample sections were deparaffinized in Xylene I and II and III (5 minutes). Then rehydrated in EtOH 100% (3 minutes), EtOH 100% (3 minutes), EtOH 95% (3 minutes), EtOH 95% (3 minutes), EtOH 70% (3 minutes). Sections were rinsed in distilled water (5 minutes). Stained in hematoxylin (6 minutes), rinsed in running tap water (20 minutes), decolorized in acid alcohol (1 - 3 seconds), rinsed well in tap water (5 minutes). The sections were counterstained in Eosin (15 seconds), dehydrated in EtOH 95% (3 minutes), EtOH 95% (3 minutes), EtOH 100 % (3 minutes), EtOH 100 % (3 minutes) cleared in Xylene I and II (5 minutes), mounted with Cytoseal in fume hood.

### Statistical analysis

All data were expressed as means ± SE. Independent-samples *t* test was used to compare the difference between two groups. One-way analysis of variance (ANOVA) followed by posthoc LSD test was used to compare the difference among multiple groups. *P* value less than 0.05 was considered statistically significant. All data were analyzed with SPSS11.5 software.

## Results

### Changes of serum albumin in cirrhotic rats before and after nutritional treatment

**Figure 2:**
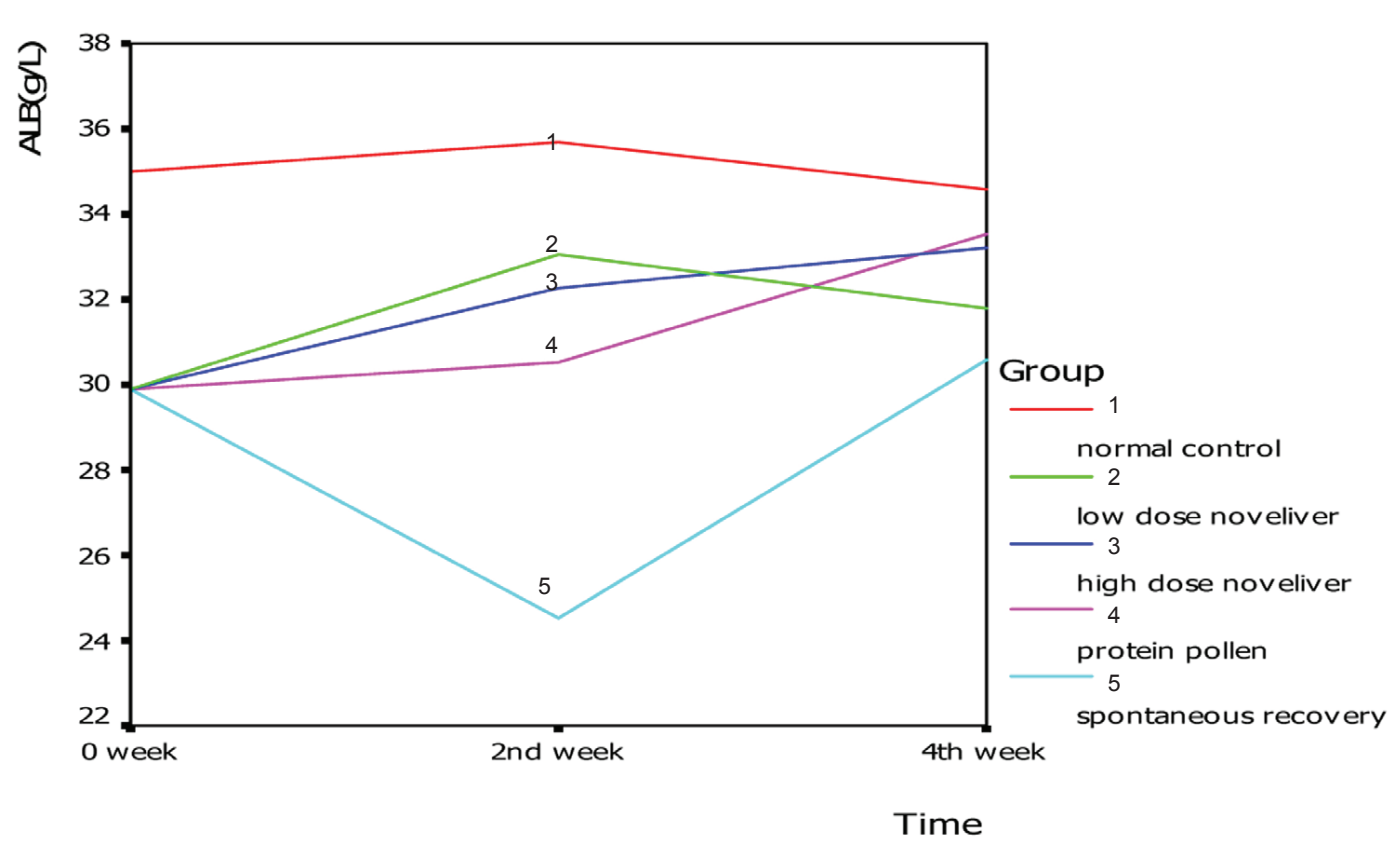
Changes of serum albumin in cirrhotic rats in 0, 2nd, 4th week of nutrition treatment.

**Figure 3:**
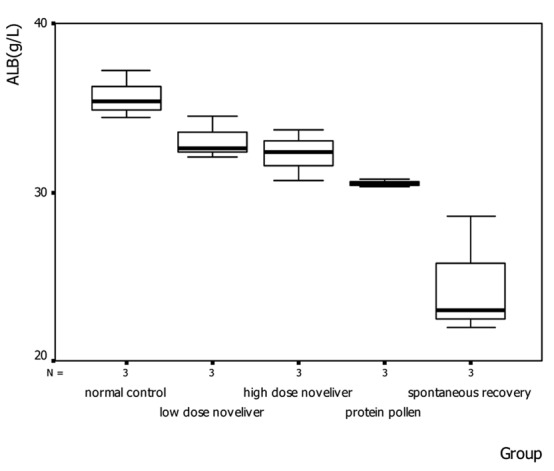
The levels of serum albumin in cirrhotic rats in the 2nd week of nutrition treatment.

In the 2nd and 4th week of nutrition treatment, the levels of serum albumin in low dose Noveliver group, high dose Noveliver group and the compound protein pollen group tended to be higher than that spontaneous recovery group (Fig. 2). In the 2nd week of nutrition treatment, the level of serum albumin in normal control group, low dose Noveliver group, high dose Noveliver group, compound protein pollen group and spontaneous recovery group were 35.67 ± 1.42, 33.07 ± 1.27, 32.27 ± 1.50, 30.53 ± 0.25, 24.53 ± 3.56 (g/L), respectively, and the difference among the groups was statistically significant, (F = 14.007, *P* = 0.000); the levels of serum albumin in low dose Noveliver group, high dose Noveliver group and the compound protein pollen group were higher than that in spontaneous recovery group (*P* = 0.000, 0.001, 0.003); the levels of serum albumin in low dose Noveliver group and high dose Noveliver group were higher than that in compound protein pollen group, but the difference was not statistically significant (*P* = 0.139, 0.297) (Fig. 3).

### Changes of liver function in cirrhotic rats before and after nutritional treatment

At the beginning of nutritional treatment, the levels of ALT, AST, TBil and DBil in cirrhotic model group tended to be higher than that in normal control group (*t* = 2.92, 2.53, 1.14, 2.34; *P* = 0.027, 0.045, 0.3, 0.058). In the 2nd and 4th week of nutritional treatment, the differences of ALT and AST among the groups were not significant (*P* > 0.05). In the 4th week of nutritional treatment, the differences of DBil and TBil among the groups were not significant (*P* > 0.05). In the 2nd week of nutritional treatment, the differences of DBil and TBil among the groups were significant (TBil: F = 4.13, *P* = 0.031; DBil: F = 3.625, *P* = 0.045), and the level of DBil and TBil in spontaneous recovery group was higher than that in the low dose Noveliver group, high dose Noveliver group and the compound protein pollen group (TBil: *P* = 0.011, 0.012, 0.019; DBil: *P* = 0.023, 0.014, 0.011) (Table 1-3).

**Table 1:** Levels of ALT, AST, TBil and DBil in Rats in the Beginning of Nutrition Treatment

	ALT (U/L)	AST (U/L)	TBil (µmol/L)	DBil (µmol/L)
Normal control group	65.4 ± 11.57	209.78 ± 38.69	3.25 ± 2.96	0.8 ± 0.42
Cirrhotic model group	156.35 ± 61.19	446.2 ± 182.65	5.57 ± 2.83	1.88 ± 0.82

**Table 2:** Levels of ALT, AST, TBil and DBil in the 2nd Week of Nutrition Treatment

	ALT (U/L)	AST (U/L)	TBil (µmol/L)	DBil (µmol/L)
Normal control group	61.17 ± 7.47	178.5 ± 41.27	1.7 ± 0.36	0.63 ± 0.15
Low dose noveliver group	63.80 ± 15.39	167.93 ± 14.75	2.47 ± 0.21	1.1 ± 0.4
High dose noveliver group	57.30 ± 10.15	173.23 ± 26.23	2.53 ± 0.74	0.8 ± 0.26
Compound protein pollen group	77.87 ± 11.05	189.6 ± 33.47	3 ± 0.17	0.7 ± 0.2
Spontaneous recovery group	71.28 ± 24.72	174.23 ± 35.32	7.48 ± 4.32	3.81 ± 2.7

**Table 3:** The levels of ALT, AST, TBil and DBil in Rats in the 4th Week of Nutrition Treatment

	ALT (U/L)	AST (U/L)	TBil (µmol/L)	DBil (µmol/L)
Normal control group	54.83 ± 15.02	223.57 ± 32.71	2 ± 0.36	1.13 ± 0.21
Low dose noveliver group	52.23 ± 6.50	161.9 ± 27.54	3.35 ± 1.77	1.5 ± 0.58
High dose noveliver group	58.7 ± 7.67	161.65 ± 41.35	2.0 ± 0.36	0.68 ± 0.10
Compound protein pollen group	74.2 ± 15.44	194.5 ± 41.06	2.2 ± 0.95	0.97 ± 0.12
Spontaneous recovery group	75.6 ± 22.97	205 ± 56.90	2.53 ± 0.38	0.93 ± 0.12

### Changes of HGF and TGF-β in cirrhotic rats before and after nutritional treatment

**Figure 4:**
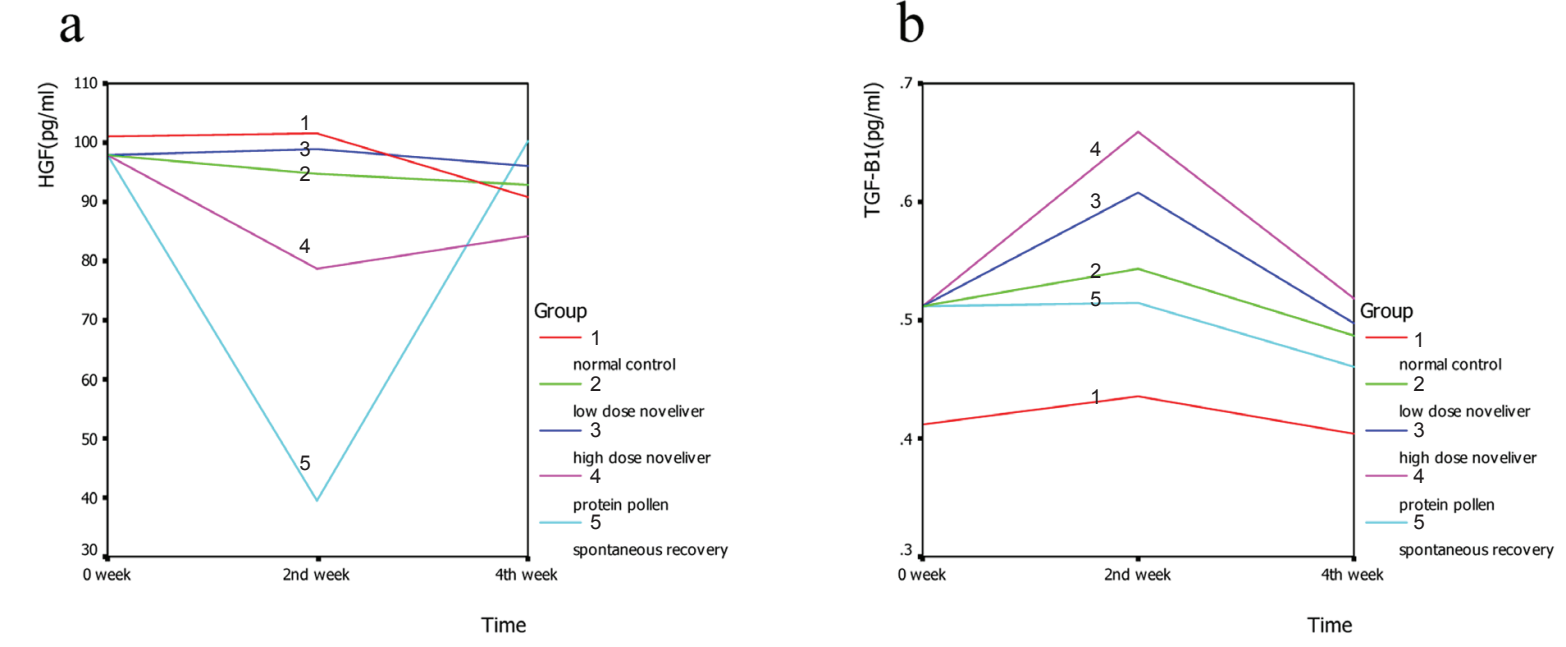
Changes of HGF (a) and TGF-β (b) in cirrhotic rats in 0, 2nd, 4th week of nutrition treatment.

In the 2nd week of nutrition treatment, the levels of HGF in normal control group, low dose Noveliver group, high dose Noveliver group, compound protein pollen group and spontaneous recovery group were 101.55 ± 0.87, 94.62 ± 8.80, 98.94 ± 3.68, 78.77 ± 21.79, 39.52 ± 14.03 (pg/ml), respectively, and the differences among the groups were significant (F = 11.12, *P* = 0.002); The levels of HGF in low dose noveliver group, high dose noveliver group and the compound protein pollen group were higher than that in the spontaneous recovery group (*P* = 0.001, 0.000, 0.005). In the 2nd week of nutrition treatment, the levels of TGF-β1 in normal control group, low dose Noveliver group, high dose Noveliver group, compound protein pollen group and spontaneous recovery group were 0.44 ± 0.14, 0.54 ± 0.07, 0.61 ± 0.14, 0.66 ± 0.03, 0.51 ± 0.03 (pg/ml), respectively, the differences among the groups were significant (F = 2.41, *P* = 0.126) (Fig. 4).

### Histology assay in cirrhotic rats before and after nutritional treatment

**Figure 5:**
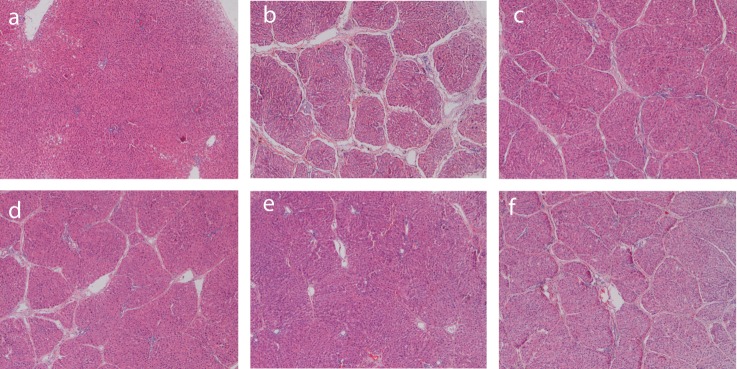
HE Staining of rat liver tissue. a: normal control group (40×); b: cirrhotic model group (40×); c: low dose Noveliver group (40×); d: compound protein pollen group (40×); e: high dose Noveliver group; f: spontaneous recovery group (40×).

In the 2nd week of nutritional treatment, fibrosis of liver tissues in all the groups was alleviated, but the differences among the groups were not apparent. In the 4th week of nutrition treatment, there were thick fibrous interval and complete pseudolobuli in cirrhosis model group; but in the low dose Noveliver group and compound protein pollen group, there were no complete pseudolobuli. In high dose Noveliver group, there were some fibers but no obvious fibrous interval. In spontaneous recovery group, there were thin fibrous interval and complete pseudolobuli. These results showed that the level of fibrosis in spontaneous recovery group was higher than that in low dose Noveliver group, high dose Noveliver group and compound protein pollen group (Fig. 5).

## Discussion

Malnutrition in patients with liver cirrhosis is quite complicated, and protein energy malnutrition is very common [14], thus nutritional support in patients with liver cirrhosis is very important. However, the choice and use of the nutrients should be considered carefully, otherwise there is a risk of increasing burden to liver [15]. In theory proteolysis in patients with liver cirrhosis is more than in normal people, so more food protein in diet is necessary to correct the negative nitrogen balance. But in clinical work, the safety range is narrow and there is significant difference in protein tolerance among cirrhotic patients. If too much protein is eaten (80 g/d), the production of toxin and ammonia will increase, which may induce hepatic encephalopathy. The purpose of plant foods is to provide rich branched-chain amino acid (BCAA) and fiber, but the intake of nutrients such as protein, calcium, magnesium, selenium, folic acid, fat soluble vitamins is insufficient [16]. Deficiency of vitamins, minerals and trace elements in patients with liver cirrhosis is common. Lack of zinc could induce liver fibrosis and hepatic encephalopathy. Lack of selenium could reduce activity of glutathione peroxidase, and affect decomposition of oxide and removal of radicals, which aggravates the injury and necrosis of liver. Lack of calcium and vitamin D in end stage liver disease will increase the incidence of osteoporosis [17]. Thus, patients with liver cirrhosis need a compound nutrition support, but food supplementation does not meet the nutritional requirements. If nutrients rich in protein, amino acid, trace elements, vitamins and minerals are added and the patients’ diet is proper, this will help to maintain liver function, enhance recovery of liver damage, and ameliorate malnutrition.

Previous studies suggested that compound nutrition support based on pollen can improve liver function, and can be better ingredients for liver nutrition [9-13]. Both Noveliver and compound protein pollen are this kind of compound nutrients. Noveliver is a new pure compound nutrient and immunomodulator for patients with liver disease, developed by Myer Otec Institute For Liver Cell Media, California, USA. It is mainly composed of the bee pollen, albumin, phospholipids and yeast rich in selenium. It is rich in all kinds of amino acid, protein, vitamins, minerals and minor elements, phospholipids, folic acid, adenosine, alkaloid, carbohydrate. It is a good formula and can provide compound nutrition for liver metabolism. Compared with compound protein pollen, Noveliver is added selenium and folic acid on the basis of the pollen, the ratio of aromatic and branched-chain amino acid is adjusted, and is designed for malnutrition and complications in end stage liver disease.

In this study, we prepared cirrhotic rat model using CCl4, pathological examiation showed pseudolobuli formation after 12 weeks, indicating that the model was prepared successfully. Then we administered two compound nutrients based on pollen to cirrhosis rats, and results showed that the levels of serum albumin in low dose Noveliver group, high dose Noveliver group and the compound protein pollen group were higher than that in spontaneous recovery group in the second week of treatment (P = 0.000, 0.001, 0.003), which indicated compound protein pollen nutrients can increase serum albumin in cirrhotic rats, and can ameliorate malnutrition in cirrhotic rats. The levels of serum albumin in low dose Noveliver group and high dose Noveliver group were inclined to be higher than that in compound protein pollen group in the second week of treatment, but the difference was not significant (P = 0.139, 0.297), which indicated that as a new liver nutrient, Noveliver may be the same as the compound protein pollen, even more advantageous in improving malnutrition. According to the results of liver function in spontaneous recovery group that the level of aminotransferase did not decrease until the 2nd week of treatment, and the level of bilirubin increased at the first week and began to decrease in the 2nd week of treatment, we presumed that cirrhotic rats induced by CCl4 had a spontaneous recovery process and liver function recovered partly in the 4th week after discontinuing CCl4 injection, which might be the reason that difference between nutritional treatment groups and spontaneous recovery group was significant in the 2nd week of treatment, but the difference in the 4th week of treatment was not significant.

Albumin is synthesized and secreted by the liver, it consists more than 60% of free serum proteins. Albumin has many important functions, such as maintaining plasma colloid osmotic pressure, anti-oxidation and substances transfer. Cirrhotic patients can be treated with injection of albumin to increase serum albumin level, but this may have side effects, such as intolerance to heterologous protein, increased risk of HIV and HCV infection, inhibiting the synthesis of albumin, and increasing the decomposition of albumin. Therefore, it is important to prompt the synthesis of albumin by improving liver function. In this study, the nutritional treatment with compound pollen protein nutrient can increase serum albumin in cirrhotic rats, in addition to the nutrients existing in the pollen protein which are essential for albumin synthesis, the liver repair and regeneration after nutritional treatment might play a more important role. The levels of HGF in low dose Noveliver group, high dose Noveliver group and the compound protein pollen group were higher than that in spontaneous recovery group in the second week of treatment (*P* = 0.001, 0.000, 0.005), this indicated that nutritional treatment may enhance liver regeneration. The histological results showed the fibrosis in nutritional treatment groups was alleviated, which may also enhance liver regeneration, the improvement of malnutrition can prompt liver repair and regeneration.

HGF plays an important role in liver regeneration. The levels of HGF in serum and liver tissue rise rapidly after partial hepatectomy, which can reach15 - 17 folds higher than normal level. Passos de Jesus et al found that proline or glutamine supplementation in malnourished rats improves total RNA content in the remnant hepatic tissue. Amino acids administration increased HGF gene expression after partial hepatectomy in malnourished rats, proline has a greater effect than glutamine [18]. TGF-β1 is a versatile cytokine, which is involved in cell proliferation and synthesis of extracellular matrix and participates in secondary DNA synthesis by HGF and fibrosis occurrence. In this study, the levels of HGF in nutritional treatment groups was higher than that in spontaneous recovery group, indicating that nutritional treatment may enhance liver regeneration.

There are some limitations in this study, the cirrhotic model induced by CCl4 had a spontaneous recovery process after discontinue of CCl4 injection, which was different from the hepatitis B related cirrhosis in terms of the course of disease. But in the period of the 2nd week after discontinue of CCl4 injection, this model can be used in the study the effect of nutritional treatment. We conclude that nutritional treatment can help liver regeneration, but the evidences might be insufficient. Next we will examine the indexes related to liver regeneration and albumin expression in different stages and we expect to explain the mechanisms of increased serum albumin in cirrhotic rats treated with compound pollen protein nutrients, this might provide new approaches for the correction of low blood albumin and protein malnutrition in cirrhotic patients.
